# Biogeochemical Control of the Coupled CO_2_–O_2_ System of the Baltic Sea: A Review of the Results of Baltic-C

**DOI:** 10.1007/s13280-013-0485-4

**Published:** 2014-01-12

**Authors:** Anders Omstedt, Christoph Humborg, Janusz Pempkowiak, Matti Perttilä, Anna Rutgersson, Bernd Schneider, Benjamin Smith

**Affiliations:** 1 Department of Earth Sciences, University of Gothenburg, Guldhedsgatan 5A, 41320 Göteborg, Sweden; 2 Department of Applied Environmental Science, Stockholm University, Svante Arrhenius väg 8, 11418 Stockholm, Sweden; 3 Institute of Oceanology of Polish Academy of Sciences, Powstancow Warszawy 55, P.O. Box 148, 81-712 Sopot, Poland; 4 Finnish Meteorological Institute, P.O. Box 503, 00101 Helsinki, Finland; 5 Department of Earth Sciences, Uppsala University, Villav. 16, 75236, Uppsala, Sweden; 6 Leibniz-Institute for Baltic Sea Research, Seestrasse 15, 18119, Warnemünde, Germany; 7 Department of Physical Geography and Ecosystem Sciences, Lund University, Geocentrum II, Sölvegatan 12, 22362 Lund, Sweden

**Keywords:** Ocean acidification, Eutrophication, Climate change, Baltic Sea, Kattegat

## Abstract

Past, present, and possible future changes in the Baltic Sea acid–base and oxygen balances were studied using different numerical experiments and a catchment–sea model system in several scenarios including business as usual, medium scenario, and the Baltic Sea Action Plan. New CO_2_ partial pressure data provided guidance for improving the marine biogeochemical model. Continuous CO_2_ and nutrient measurements with high temporal resolution helped disentangle the biogeochemical processes. These data and modeling indicate that traditional understandings of the nutrient availability–organic matter production relationship do not necessarily apply to the Baltic Sea. Modeling indicates that increased nutrient loads will not inhibit future Baltic Sea acidification; instead, increased mineralization and biological production will amplify the seasonal surface pH cycle. The direction and magnitude of future pH changes are mainly controlled by atmospheric CO_2_ concentration. Apart from decreasing pH, we project a decreasing calcium carbonate saturation state and increasing hypoxic area.

## Introduction

**Fig. 1:**
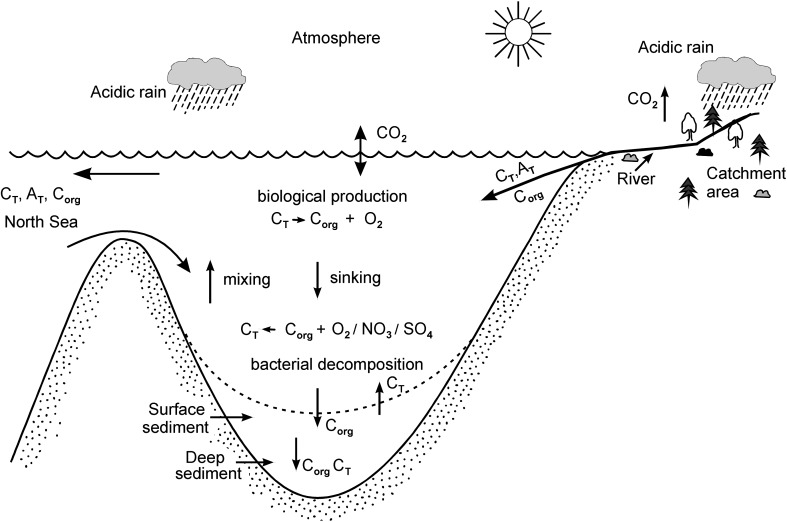
Schematic of the Baltic Sea carbon cycle: C_org_, organic carbon; C_T_, total inorganic carbon; A_T_, total alkalinity; CO_2_, carbon dioxide (redrawn from Omstedt et al. 2009 and including mineralization trough oxygen/nitrate/sulfate (O_2_/NO_3_/SO_4_) reduction)

Coastal seas, such as the Baltic Sea, link continents and oceans via freshwater and matter fluxes. However, before the supplied chemical substances reach the ocean, they undergo alternations that depend on physical, chemical, and biological conditions in the coastal seas. Changes due to eutrophication, deoxygenation, marine acidification, and climate change may also severely affect the carbon and nutrient cycles and therefore the marine ecosystems. CO_2_ and O_2_ dynamics (Fig. 1) are central to these changes in the marine biogeochemical cycles and for the ecosystem health.

Commonly, the term eutrophication is related to the excessive nutrient load of a sea area. A more appropriate definition considers eutrophication as “an increase in the rate of supply of organic matter to an ecosystem” (Nixon 1995). This definition is based on the fact that organic matter is a major control for the marine food web and oxygen depletion in deeper water layers. Hence, the modeling of eutrophication requires the explicit involvement of carbon as state variable that is no longer linked to the nutrient consumption by the traditional Redfield ratio (Redfield et al. 1963). Furthermore, the central role of carbon also implies that the input of organic carbon from the catchment must be included in the modeling. On the other hand, any production/mineralization of organic carbon affects not only the oxygen conditions but is also directly connected with the consumption/release of CO_2_. Including these processes in biogeochemical models and taking into account the input of inorganic carbon from land facilitate the simulation of the Baltic Sea acid–base system and thus address the marine acidification caused by increasing atmospheric CO_2_. Based on these considerations, a model framework was developed and complemented by measurements and data analysis that accounted for processes both in the sea and in the catchment which are relevant for the Baltic Sea O_2_–CO_2_ system (Fig. 1).

Eutrophication, acidification, and climate change are connected through the primary production and mineralization of organic matter from the sea and land. The coupling is complex, involving interconnection between organisms throughout the drainage basin and human activity. Human actions directly influence the carbon and nutrient cycles and may cause severe damage. Scientific knowledge, improved monitoring, and developing models addressing the carbon and nutrient cycles are therefore essential. These were addressed by BONUS+ in the Baltic-C program (http://www.baltex-research.eu/baltic-c/), where extensive fieldwork, database development, and modeling were the main activities during a 3-year research program starting in 2009. Baltic-C science was based on interdisciplinary cooperation among scientists from seven institutions and four countries. The present paper reviews some of the Baltic-C findings.

### Experimental Studies

Biogeochemical models are based on mathematical process descriptions that include various empirical parameters. Due to the complexity of biogeochemical processes, these parameterizations only crudely approximate reality and are not universal laws applicable to all marine ecosystems. This particularly refers to the brackish Baltic Sea with its special hydrographic characteristics, permanent anoxic areas, and exposure to nutrients and carbon inputs from its catchment. Baltic-C biogeochemical modeling was therefore supported by a comprehensive measurement program and by monitoring data analysis to improve process parameterizations and provide model validation data (Leinweber et al. 2005; Kuznetsov et al. 2011). Activities focused on the marine CO_2_–O_2_ system, since almost all biogeochemical transformations entail CO_2_–O_2_ consumption or release. This also implies that the biogeochemical modeling was evaluated for its ability to simulate seasonal and spatial variations in the marine CO_2_–O_2_ system.

### Relationship Between Surface Water pCO_2_ and Net Community Production

An automated measurement system for determining CO_2_ partial pressure (pCO_2_) was deployed on a cargo ship to investigate the seasonality and spatial distribution of surface water pCO_2_ (Schneider et al. 2006). The ship commutes 2–3 times per week between Luebeck in the southwest and Helsinki in the northeast Baltic Sea. This corresponds to a mean temporal resolution of the data acquisition of about 2 days. The spatial resolution given by ship speed and the measurement system response time was 1–2 nautical miles. Measurements were made with the Finnish Alga line Project, which records chlorophyll fluorescence and automatically samples surface water for nutrient analysis. pCO_2_ measurements started in summer 2003, stopped for 1.5 years when another ship took over the Luebeck–Helsinki route, and resumed in the long-term observation program of the Baltic Sea Research Institute (IOW, Warnemuende). For particular years and seasons, the data were used to estimate production and nitrogen fixation (Schneider et al. 2006, 2009). We present an overview of the data and draw conclusions regarding the seasonality of net community production and its relationship with nutrient availability following the analytical methods given by Koroleff (1983).

**Fig. 2:**
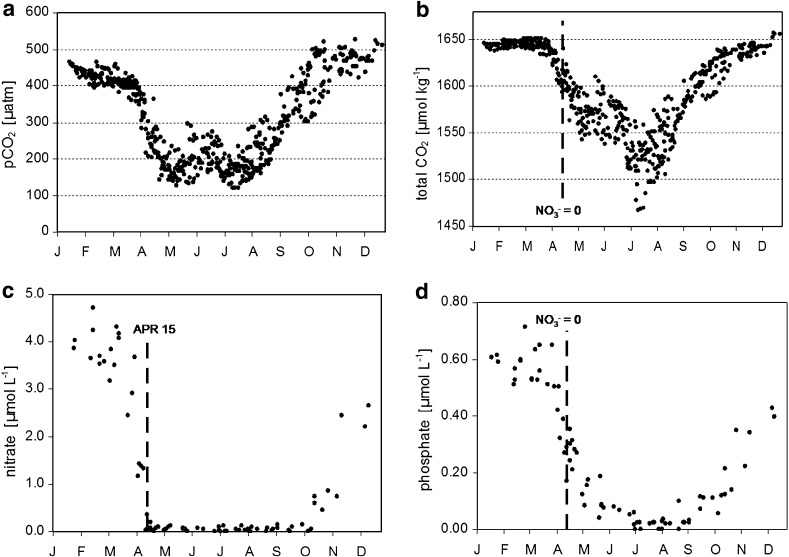
**a** Seasonality of the CO_2_ partial pressure, **b** total CO_2_, **c** nitrate, and **d** phosphate in the eastern Gotland Sea, 2004–2011 (data from measurements and sampling on VOS Finnpartner-Finnmaid)

Figure 2a shows the seasonality of mean pCO_2_ in the northeastern Gotland Sea (57.5°–58.5°), 2004–2011. From April to about October, the pCO_2_ was clearly below atmospheric pCO_2_, which was 385–400 μatm in these years. This indicates that CO_2_ consumption by biological processes controlled pCO_2_ in this period, dominating the effect of rising temperatures in spring and summer that would increase pCO_2_. The seasonal pCO_2_ distribution is characterized by the two minima observed in spring and mid-summer, resulting from interplay between production peaks and increasing temperatures. The pCO_2_ increase after the main productive period coincides with the deepening of the mixed layer transporting CO_2_-enriched water masses to the surface. This process causes oversaturation of the surface water relative to atmospheric CO_2_, so CO_2_ is released into the atmosphere from November to March.

Based on pCO_2_ data, seasonal changes in total CO_2_ (C_T_) were calculated, which together with estimated CO_2_ gas exchange yield the net community production (NCP). Calculations were facilitated by the virtual absence of calcifying plankton from the central Baltic Sea (Tyrrell et al. 2008), so internal alkalinity changes were negligible. The mean total alkalinity in the northeastern Gotland Sea could be used to calculate seasonal C_T_ changes from the pCO_2_, temperature and salinity data (Schneider et al. 2009).

**Fig. 3:**
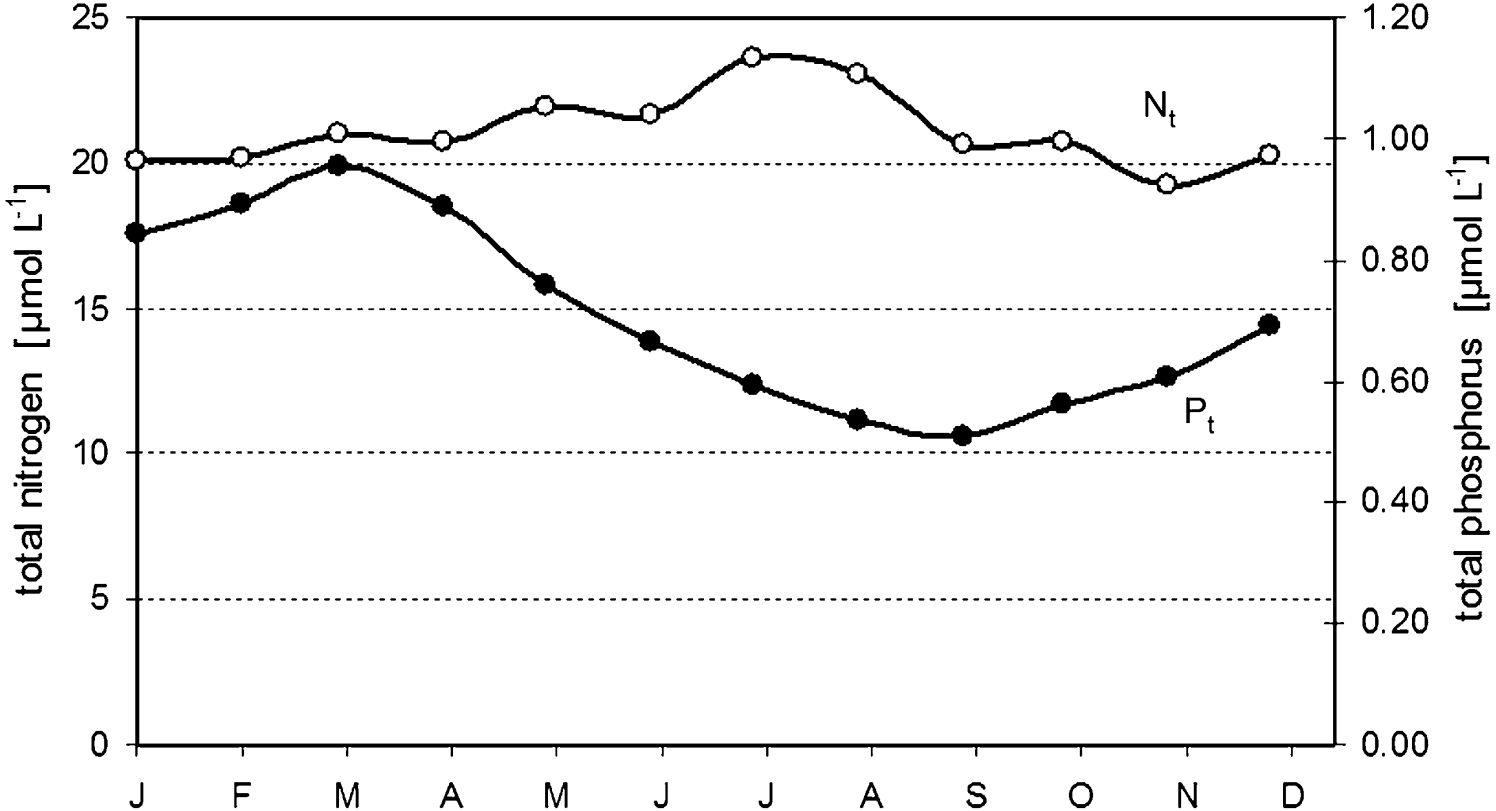
Mean seasonality of total nitrogen (N_t_) and total phosphorus (P_t_) in the eastern Gotland Sea, 2004–2010 (data from the Swedish National Monitoring Program, SMHI)

The sharp C_T_ drop occurring in all years in almost the same week by the end of March indicated the start of spring phytoplankton bloom (Fig. 2b). The C_T_ decreased until mid-May, although nitrate was already entirely depleted in all years by mid-April (Fig. 2c). This indicates the continuation of net community production, since most excess phosphate left after nitrate depletion was concurrently consumed (Fig. 2d). This raises the question of the nitrogen source required for this production. Nitrate input from vertical mixing can be excluded, as nitrate is entirely exhausted to depths of 50–60 m after early spring bloom, while the mixed layer is only 20–30 m deep in the post-nitrate production period. Likewise, lateral transport cannot occur because nitrate concentrations in the surface water of the entire Baltic Proper, including coastal areas, are nearly zero. It has been speculated that nitrogen is preferentially mineralized and transferred from the existing biomass pool to new production using the phosphate excess after nitrate depletion (Thomas et al. 1999). This implies that the mean N/P ratio of the produced organic matter (POM) must approximately correspond to the low winter nitrate/phosphate ratio which on average is about 8 (Nausch et al. 2008). However, measurements yielded N/P ratios in POM close to or even above the Redfield ratio (16) during spring (Schneider et al. 2003). This indicates that preferential nitrogen mineralization plays only a minor role and cannot explain the continuation of net community production after nitrate depletion. Hence, another nitrogen source must exist; since atmospheric deposition is far too small to cause short-term effects, we speculated that either dissolved organic nitrogen was used for production or early nitrogen fixation took place despite low water temperatures in late April and early May. However, neither hypothesis could be substantiated by field measurements. But the analysis of monitoring data for total nitrogen and phosphorus concentrations in the eastern Gotland Sea (Swedish National Monitoring Program, SMHI) indicated that total nitrogen increased after the nitrate depletion, while total phosphorus decreased continuously from the start of spring bloom due to sedimentation (Fig. 3). These findings suggest an external nitrogen source, such as nitrogen fixation.

From mid-May to mid-June, C_T_ did not display a clear trend. This indicates that net organic matter production was small and that the biological activity was based on regenerated production during which nutrients are recycled in the trophic layer. Since the post-nitrate bloom differed considerably between years, the ensuing regenerated production started at different C_T_ levels causing the broad C_T_ range in this phase (Fig. 2b).

A second distinct drop in C_T_ was observed by mid-June when the well-documented mid-summer production based on nitrogen fixation started. The minimum generally occurred in July and indicated strong interannual variation in the minimum levels, which do not necessarily reflect variations in integrated production and nitrogen fixation in the trophic layer. The C_T_ minima were confined to a shallow water layer about 2–3 m deep and occurred only during extremely calm weather conditions that produced high temperatures at the water surface. Finally, the phosphorus supply for production during nitrogen fixation must be considered. Excess phosphate was almost completely consumed by the mid-June start of nitrogen fixation. Furthermore, the continuous decrease in total phosphorus (Fig. 3) indicates that this phosphate was widely removed from the surface and was no longer a significant source of production during nitrogen fixation. Although it has been speculated that dissolved organic phosphorus and/or upwelling events (Nausch et al. 2009) may provide phosphorus for production, there is clearly a phosphorus shortage in the nitrogen fixation period. The lack of phosphorus obviously does not limit nitrogen fixation, and organic matter production results in C/P and N/P ratios that may exceed the corresponding Redfield ratios by a factor of up to four (Larsson et al. 2001; Schneider et al. 2003).

### Deep Water Carbon Mineralization and Carbon Burial in the Sediment

**Fig. 4:**
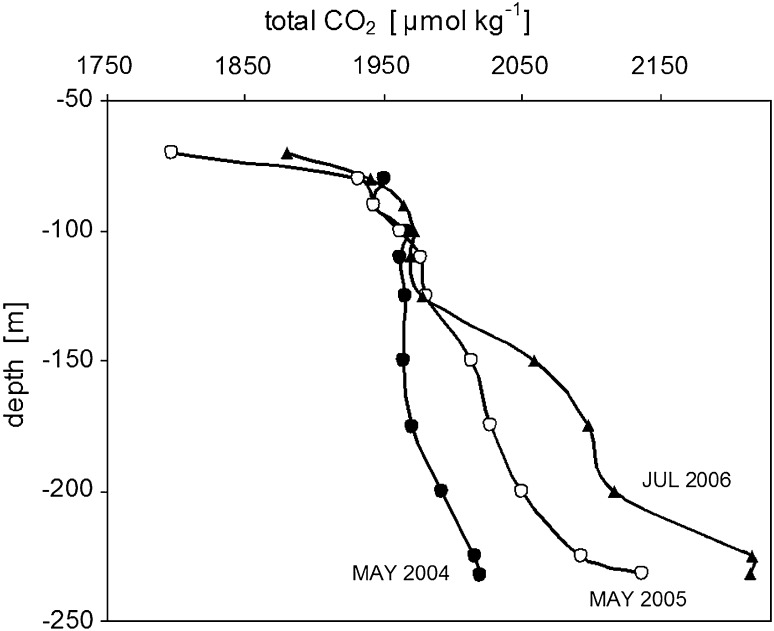
Vertical profiles of total CO_2_ in the eastern Gotland Sea during a stagnation period that lasted from May 2004 to July 2006

To support Baltic-C modeling of organic matter mineralization, deep water total CO_2_ data were analyzed in Baltic-C. The measurements were made as part of the IOW’s long-term observation program. C_T_ profiles were measured five times per year at the central station (BY15) in the eastern Gotland Sea. The vertical resolution was 25 m in the deeper part of the basin. From May 2004 to July 2006, temperature and salinity distributions indicated almost ideal stagnant conditions in the water masses below 150 m. The basin could thus be considered a biogeochemical reaction vessel, i.e., a closed system, unaffected by lateral water exchange, and could be used to trace the kinetics of biogeochemical transformation related to organic matter mineralization. C_T_ accumulation below 150 m during the beginning, middle, and end of the stagnation period is shown in Fig. 4. Based on a mass balance accounting of the vertical exchange, the C_T_ increase was used to calculate mineralization rates for different depth intervals (Schneider et al. 2010). We found that mineralization occurred mainly at the sediment surface and that the rates did not depend on redox conditions. The mean mineralization rate for the area below 150 m was 2.0 mol-C m^−2^ year^−1^, consistent with a previous finding of 1.8 mol-C m^−2^ year^−1^ (Schneider et al. 2002). However, our value is higher than that of Gustafsson and Stigebrandt (2007), who reported a rate of 1.3 mol-C m^−2^ year^−1^ based on oxygen and hydrogen sulfide data from 14 stagnation periods between 1965 and 2004. These estimates refer to different years and time spans which may differ with regard to the organic matter input. This may partly explain the differences in the calculated mineralization rates.

Not all organic material entering the deep basins is mineralized. A significant fraction of the organic matter either produced in the sea or transported to the sea from land is deposited to the sediments. Substantial portion of the deposited organic matter is mineralized, and defuses back to the overlaying water. The unmineralized fraction is permanently buried in sediments. Preliminary estimates indicate that the amount of organic carbon buried in sediment is about 2.7 Tg-C year^−1^ (0.58 mol-C m^−2^ year^−1^) (Kuliński and Pempkowiak 2011). However, these burial rates are associated with considerable uncertainty due to the limited data on both sediment accumulation rates and the range of organic carbon concentrations in bottom sediments.

Profiles of organic carbon concentrations in bottom sediments of the Baltic Sea indicate that organic matter concentrations decrease with sediment depth (Emeis et al. 2000; Szczepańska et al. 2012). This is attributed to the recent increased organic matter deposition caused by eutrophication (Emeis et al. 2000; Voss et al. 2000) and to ongoing mineralization of labile organic matter in deeper sediment layers (Kuliński and Pempkowiak 2011; Szczepańska et al. 2012). The mineralization occurs in two stages: the first lasting some 10 years, and the second lasting 50–60 years (Kuliński and Pempkowiak 2011).

Organic carbon burial in bottom sediments of the Baltic Sea was calculated as the difference between organic carbon accumulated in deep depositional areas of the Baltic Sea and organic matter losses due to long-term mineralization and diffusion into the water column. Carbon accumulation rates were determined from sediment accumulation rates based on the ^210^Pb method and validated against the ^137^Cs distribution (Pempkowiak 1991; Szczepańska et al. 2012) and from organic carbon concentrations in the sediments. Carbon losses caused by long-term mineralization were calculated from concentrations of inorganic carbon dissolved in pore water and from diffusion into the water column, where they contribute to accumulated total CO_2_. Likewise, the profiles of dissolved organic matter in pore water yielded the reflux into the water column. The details of organic carbon burial rate quantification in Baltic Sea sediments have been described by Kuliński and Pempkowiak (2011) and Szczepanska et al. (2012). Based on 23 sediment cores, the carbon accumulation, burial, and reflux rates were determined for the major depositional areas of the Baltic Sea (Table 1). The differences in burial rates between the basins are large. The highest rate observed in the Gotland Basin is 3.5 times larger than that in the Gulf of Bothnia, partly due to the lower productivity in the Gulf of Bothnia, and also due to the high lateral organic matter input into the Gotland Basin (Schneider et al. 2009).

**Table 1:** Annual deposition of carbon to bottom sediments, return flux of organic and inorganic carbon to the overlying water, and carbon burial (in flux units and percentage of deposition). Data for the Gulf of Finland are from Algesten et al. (2006)

Study area	Deposition to sediments (mol-C m^−2^ year^−1^)	Return flux (DIC and DOC) (mol-C m^−2^ year^−1^)	Carbon burial rate (mol-C m^−2^ year^−1^)	Carbon burial (%)
Gdansk Deep	1.53 ± 0.34	1.00 ± 0.09	0.53	34
Gotland Basin	2.08 ± 0.75	0.68 ± 0.06	1.40	66
Bornholm Basin	1.67 ± 0.10	0.85 ± 0.06	0.82	49
Gulf of Bothnia	0.83 ± 0.25	0.43 ± 0.02	0.40	48
Gulf of Finland	1.92 ± 0.25	0.84	1.08	59

## Baltic Basin Modeling

### Baltic-C Modeling System

The Baltic-C program developed and applied a new land–sea carbon model system for the Baltic Sea and drainage basin. The model system involves two land surface models, i.e., LPJ-GUESS (Lund-Potsdam-Jena General Ecosystem Simulator) and CSIM (Catchment Simulation Model), and one Baltic Sea model, i.e., PROBE-Baltic (Baltic Sea model applying the general equation solver PROBE, see Omstedt et al. 2012 for discussion of the models and scenarios). These models have been validated for several periods under present and past climate conditions. All three models were forced using downscaled climate data according to the chosen scenario narratives. Selected IPCC-SRES narratives (i.e., A2, A1B, and B1), together with climate model simulations based on them, were used as the basic scenario framework of this study. A1B corresponds to a story line with rapid global economic growth, with a mid-twenty-first century peak in fossil fuel emissions and with atmospheric CO_2_ concentrations up to 700 μatm. A2 corresponds to a story line with a heterogeneous world with slow technological development and continuously increasing fossil fuel emissions up to 850 μatm, while B1 corresponds to a convergent world with a focus on global sustainability and atmospheric CO_2_ concentrations stabilizing at 550 μatm. Twelve Global Climate Model (GCM) scenarios, downscaled for the Baltic Sea Basin using the RCA3 (Rossby Centre regional climate Atmosphere model, version 3), were chosen to encompass the possible future climate development of the twenty-first century and to accommodate uncertainties regarding the global climate system (represented by three GCMs), natural climate variability (represented by three ensemble members of the ECHAM5 GCM, European Centre model, Hamburg version no 5), and future social and economic development (represented by three greenhouse gases emission scenarios). Three scenario runs started from ECHAM5 but use different land cover assumptions and nutrient loads. One of these runs was defined as business as usual using A2 emissions (BAU-A2), a second run was defined as a medium scenario (medium-A1B), and a third, and most optimistic run used nutrient loads according to the Baltic Sea Action Plan (BSAP; HELCOM 2007) and the B1 emission scenario (BSAP-B1). Due to severe biases in the GCM’s water and heat balances, bias corrections were introduced.

### Modeling the Baltic Sea’s Past and Present CO_2_–O_2_ System

**Fig. 5:**
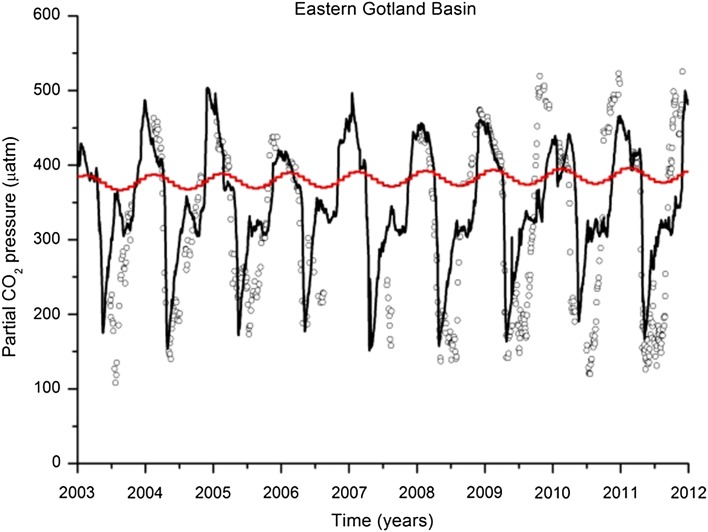
Surface water partial CO_2_ pressure from observations (*circles*) and from the model calculations (*line*, PROBE-Baltic version 3.0, Omstedt et al. 2012). The observations represent measurements from VOS Finnpartner-Finnmaid also illustrated in Fig. 2a. The *red curve* indicates the partial pressure in the atmosphere

To investigate the Baltic Sea CO_2_–O_2_ system, Omstedt et al. (2009) developed a fully coupled physical–biogeochemical model of CO_2_ uptake and release. The model included interaction between physical (i.e., currents, turbulent mixing, stratification, temperature, salinity, sun penetration, and ice), chemical (i.e., total alkalinity, pH, total CO_2_, oxygen, and nutrients), and biological (i.e., organic matter production and degradation) processes. These processes were built into an advanced process-oriented coupled basin model that has been extensively explored and validated for the Baltic Sea (Omstedt 2011). Omstedt et al. (2009) found that the long-term values of the water partial pressure of CO_2_ were above atmospheric values before industrialization, with a net release of CO_2_ to the atmosphere. Seasonal variability increased in the modern industrialization era with the inclusion of eutrophication, making the Baltic Sea both a sink and source of CO_2_ to the atmosphere. During the Baltic-C program the Baltic Sea modeling was extended by letting the mineralization raters be directly coupled to the amount of organic material in the water column and on the sea floor and expanding the biological modeling (Gustafsson 2012). Also the CO_2_–O_2_ dynamics under both oxic and anoxic conditions were introduced in the Baltic Sea model (Edman and Omstedt 2013). The modeled partial pressure of CO_2_ (Fig. 5) displays reasonable agreement with observations. The decrease during spring bloom and the increase during autumn deep water mixing are realistically modeled. However, the mid-summer minimum is missing in the model simulations. The reason is that phosphorus was consumed during the spring bloom and no longer available for the mid-summer nitrogen fixation period. As the source and the phosphorus demand for cyanobacteria are not yet identified this has not been introduced in the present model version (PROBE-Baltic version 3.0).

Factors influencing the acid–base balance were analyzed in Omstedt et al. (2010). Using calculations based on the marine carbon system and on modeling, the sensitivity of Baltic Sea surface pH was examined. This sensitivity study yielded several important insights such as:

1. Climate changes in temperature or salinity will only marginally affect the acid–base balance.
2. The direct effect on seawater pH of acid precipitation over the Baltic Sea surface was demonstrated to be small.
3. Acidification due to river transport of dissolved organic carbon into the marine system seems marginal, although mineralization of terrestrial organic carbon may cause extra marine acidification, but the effect has yet to be quantified.
4. Increased nutrient load may amplify the seasonal pH cycle, increasing acidification in winter.
5. Fossil fuel burning is likely to have both direct and indirect effects by increasing CO_2_ levels, altering seawater pH, and changing river chemistry.

### Future Changes in the Land Transport to the Baltic Sea

The Baltic-C modeling system (Omstedt et al. 2012) was applied to study possible future changes. In the scenarios simulations assuming different possible story lines from 1961 to 2100, the results from the land surface models indicated generally increased riverine fluxes, i.e., A_T_^terr^, C_T_^terr^, and C_org_^terr^. The increased riverine fluxes were largest in the northern catchments, where the increases were 20–50 %, with the largest increase in the Gulf of Finland. The increasing fluxes resulted mainly from increasing runoff, since modeled concentration changes were relatively small (<10 %). In addition, the model projected increasing fluxes in the Kattegat but no significant flux changes in the Baltic Proper. For the Danish Straits, only the modeled A_T_^terr^ increase was significant.

In the two northernmost catchments (i.e., the Bothnian Bay and Bothnian Sea), where C_org_^terr^ is the dominant carbon fraction, the modeled increase in riverine C_org_^terr^ concentration was greater than the increase in the inorganic fractions. This was especially true for the Bothnian Bay in the A2 scenario, in which the large C_org_^terr^ increase contributed to a decrease in A_T_^terr^ concentration. For the Baltic Proper and Gulf of Riga, where the inorganic fractions dominate the C_org_^terr^ fractions, the A_T_^terr^ concentration increased more than did the C_org_^terr^ concentration. The modeled scenarios suggest no decreasing riverine fluxes and very few decreasing concentrations.

### Future Changes in the Baltic CO_2_–O_2_ System

**Fig. 6:**
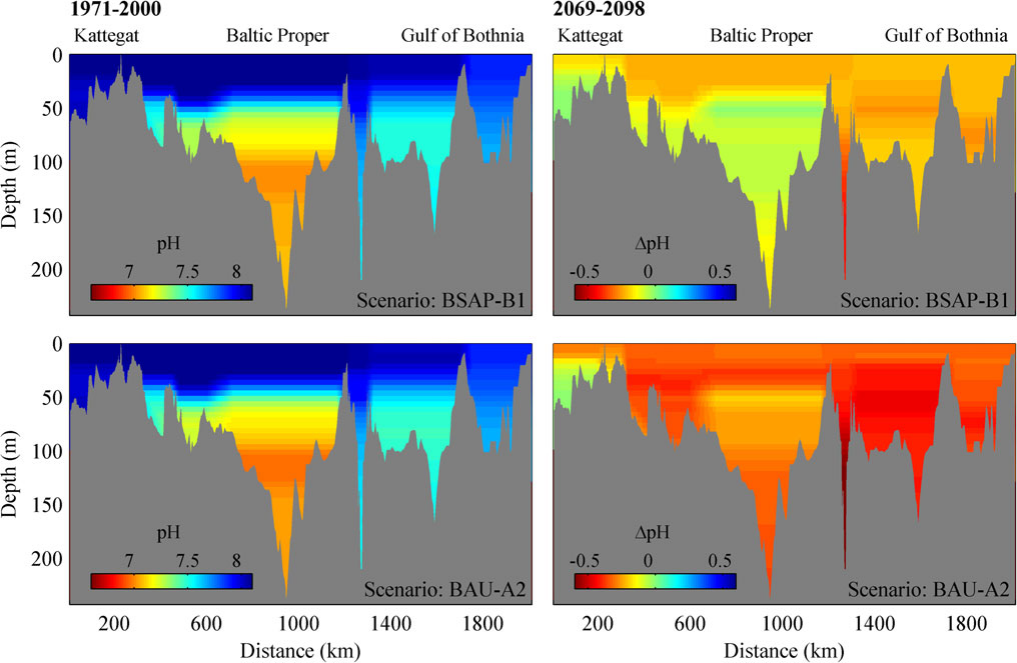
Current pH (1971–2000) and scenario pH changes (2069–2098) along a Baltic Sea transect for the BSAP-B1 (nutrient loads according to the Baltic Sea Action Plan and the B1 green house gases emission scenario) and BAU-A2 (Business As Usual and the A2 green house gases emission scenario). Figure from Omstedt et al. (2012)

**Fig. 7:**
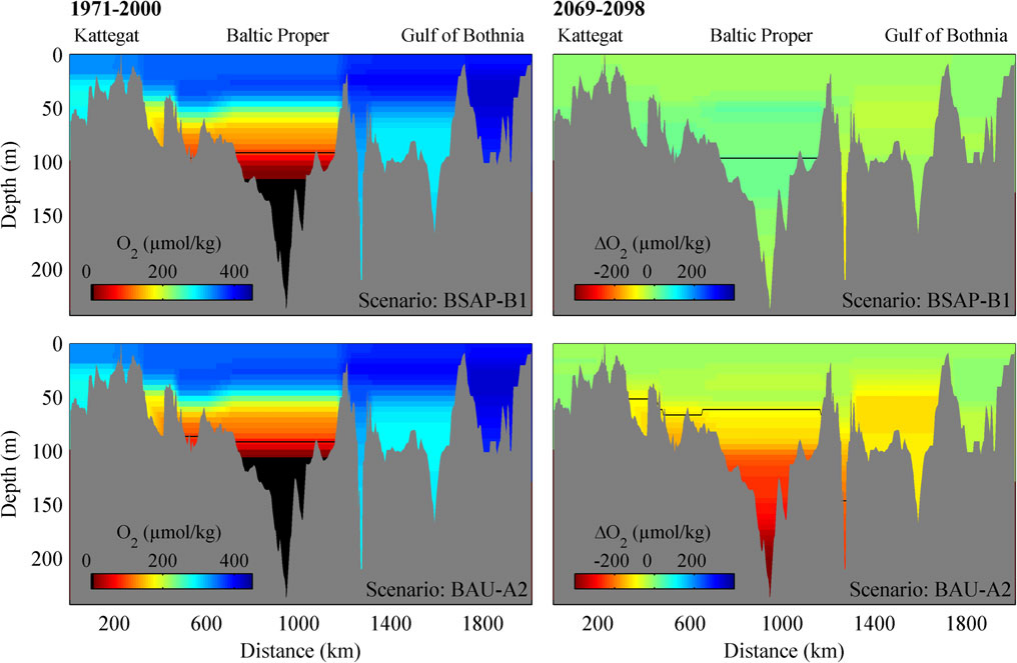
Current O_2_ (1971–2000) and scenario O_2_ changes (2069–2098) along a Baltic Sea transect for the BSAP-B1 (nutrient loads according to the Baltic Sea Action Plan and the B1 green house gases emission scenario) and BAU-A2 (Business As Usual and the A2 green house gases emission scenario). The limit for hypoxic water (set as 90 μmol kg^−1^) is indicated by the *black line*. Figure from Omstedt et al. (2012)

The scenario response of pH along a longitudinal Baltic Sea transect is illustrated in Fig. 6. The figures show the current state and the changes resulting from the BSAP-B1 and BAU-A2 scenario narratives. The results indicate that acidification will occur at most depths in both BSAP-B1 and BAU-A2, the most pronounced pH drops occurring in the surface waters, Åland Sea deep water, and intermediate or deep waters of the northern basins. The small pH variation in Kattegat deep water is due to the lateral conditions in the model, which assume constant values in the deeper parts of the Kattegat. Assuming constant lateral conditions in the deep part of the Kattegat have only minor effects on the model solution for the Baltic Sea as these levels are far below the sill depths and could be ignored. In both BSAP-B1 and BAU-A2, Baltic Proper deep water is the least affected by acidification. BSAP-B1 causes only minor changes in the oxygen concentrations in the Baltic Sea as a whole, with increasing oxygen concentrations in the deeper parts of the Baltic Proper (Fig. 7) illustrating improved water quality. These increases are caused by reduced hypoxic and anoxic conditions during stagnation periods due to lower nutrient concentrations.

In the BAU-A2 scenario, the most pronounced reductions in oxygen concentration occur in the intermediate and deep layers in the Baltic Proper, Åland Sea, and Bothnian Sea. In the Baltic Proper, the change is caused by a growing anoxic water volume, which shifts the redox cline upward, and by increased negative oxygen in the deepest volume. The oxygen reductions in the Bothnian Sea will not cause hypoxic conditions, but the volume will be deprived of almost half its oxygen content (−150 μmol kg^−1^). The reduced pH decrease in the Baltic Proper bottom-water volume is caused by the interaction between the O_2_ and CO_2_ systems. In BSAP-B1, the bottom-water pH began to level out early due to lessened CO_2_ deep water accumulation, caused by the nutrient reductions in the narrative. Continued acidification from CO_2_ emissions in BSAP-B1 is balanced by the recovery of deoxygenated water volumes until the emission signal also levels out. In BAU-A2, acidification prevails throughout the modeled period; however, the effect is somewhat counteracted in anoxic bottom waters by total alkalinity generation due to denitrification and sulfate reduction (Edman and Omstedt 2013), which dampens the effect of increased CO_2_ accumulation. The result is net acidification in Baltic Proper bottom water as well as in BAU-A2, though the pH decrease is less pronounced than in the surface waters.

## Discussion and Future Outlook

The Baltic Sea is under strong human pressure and it is critical to understand several complex processes that interact with the ecosystem. Key pressures such as eutrophication, climate change, and marine acidification all influence the Baltic Sea. Future changes will influence various processes and drivers, including changes in heat, water, nutrient, and carbon components that may strongly influence the marine ecosystem.

Observations of the Baltic Sea constitute the basis of our understanding, and new data, for example, on pCO_2_, guide us in improving biogeochemical models. Model simulations can only succeed if accompanied by measurements, which provide validation data necessary for identifying shortcomings in the process parameterization. Continuous CO_2_ and nutrient measurements with a high temporal resolution made along VOS lines are ideal for disentangling the complex biogeochemical processes in Baltic Sea surface water. Based on these data and on modeling, it is clear that standard concepts of the relationship between nutrient availability and organic matter production (i.e., the Redfield hypothesis) do not necessarily apply to the Baltic Sea. We still lack crucial knowledge; for example, we do not understand the nitrogen supply of post-nitrate net community production. In addition, our knowledge of the importance of nitrogen fixation and its controlling factors is still poor.

Present Baltic-C work also examines possible future changes in the Baltic Sea acid–base (pH) and oxygen balances. Results indicate that increased nutrient loads will not inhibit future Baltic Sea acidification, but that increased biological production and mineralization will amplify the seasonal pH cycle. CO_2_ levels in the atmosphere will likely increase in coming decades and climate warming will likely continue, with several heat balance-related implications. Increasing temperatures will also influence the water balance through changes in precipitation and evaporation. In the Baltic Sea drainage basin, we expect more precipitation in the north and less in the south, which may greatly affect salinity and biogeochemical cycles. Changes in nutrient cycles are largely due to agricultural and food consumption developments, which may increase nutrient loads in the future. Increased temperatures and CO_2_ concentrations will also change the carbon cycle, increasing the land–sea transport of organic carbon. Future expected anthropogenic climate changes in heat, water, nutrient, and carbon cycles indicate increased threats to marine ecosystems, implying a great need for management efforts related to both regional nutrient and global CO_2_ emission reductions.
